# Development of a model for modulus of polymer halloysite nanotube nanocomposites by the interphase zones around dispersed and networked nanotubes

**DOI:** 10.1038/s41598-022-06465-4

**Published:** 2022-02-14

**Authors:** Yasser Zare, Kyong Yop Rhee

**Affiliations:** 1 Biomaterials and Tissue Engineering Research Group, Department of Interdisciplinary Technologies, Breast Cancer Research Center, Motamed Cancer Institute, ACECR, Tehran, Iran; 2 Department of Mechanical Engineering (BK21 Four), College of Engineering, Kyung Hee University, 1 Seocheon, Giheung, Yongin, Gyeonggi 449-701 Republic of Korea

**Keywords:** Engineering, Materials science

## Abstract

Theoretical studies on the mechanical properties of halloysite nanotube (HNT)-based nanocomposites have neglected the HNT network and interphase section, despite the fact that the network and interphase have significant stiffening efficiencies. In the present study, the advanced Takayanagi equation for determining the modulus of nanocomposites is further developed by considering the interphase zones around the dispersed and networked HNTs above percolation onset. Furthermore, simple equations are provided to determine the percolation onset of HNTs and the volume portions of HNTs and interphase section in the network. The experimental values obtained for many samples and the assessments of all relevant factors validate the proposed model. The high ranges of HNT concentration, interphase depth, HNT modulus, HNT length, network modulus, interphase modulus, interphase concentration, and network fraction enhance the system modulus. However, the low levels of HNT radius, percolation onset, and matrix modulus can intensify the reinforcing effect. Notably, the moduli of the dispersed HNTs and the surrounding interphase negligibly affect the modulus of the samples. Moreover, HNTs cannot reinforce the polymer medium when the HNT volume fraction is lower than 0.01 and the interphase depth is less than 5 nm.

## Introduction

Halloysite nanotubes (HNTs) are alumino-silicate products extracted from natural deposits and designed from amorphous allophone^1–11^. HNTs have the tubular structure of rolled sheets owing to the forces between aluminum oxide and silicon dioxide. However, because of their large surface area and unique shape, HNTs are never linked with each other, which facilitate their hydrogen bonding with polymer media. For this reason, HNTs are easily dispersed within polymer media, and HNT-reinforced polymer nanocomposites exhibit improved mechanical, thermal, and fire-retardant properties^12–15^. HNTs increase the strength of polymers without reducing their ductility, unlike carbon nanotubes (CNTs) and nanoclays. Furthermore, HNTs are biocompatible, and polymer HNT nanocomposites offer multiple advantages such as excellent biocompatibility and considerable mechanical performance, which are necessary in advanced applications^16,17^.

Many researchers have experimentally examined the performance of polymer HNT systems^4,18,19^. Krishnaiah et al*.*^20,21^ adapted the surface of HNTs with 3-aminopropyltriethoxysilane to enrich the surface communication of HNTs with polylactide and polypropylene. Prashantha et al*.*^22^ used neat HNTs and HNTs treated with quaternary ammonium salt to fabricate polypropylene nanocomposites. Their results indicated that the modified HNTs exhibited better performance than the unmodified HNTs owing to the strong interface between the polymer medium and the modified HNTs. However, the existing models for calculating the properties of polymer HNT systems are restricted. Researchers have focused on investigating these materials experimentally. There are a few studies on the mechanical characteristics of HNT nanocomposites^17,23,24^. Several authors have used old models to determine the tensile performance of polylactide/HNT samples^17^. However, it is necessary to develop new advanced for understanding and optimizing the performance of these materials in progressive applications.

Tubular nanoparticles such as CNTs easily form networks in nanocomposites above the percolation onset^25,26^. Long and narrow nanofillers provide an extremely low percolation onset and facilitate network creation in the system at low filler contents. Therefore, long nanofillers are attractive for use in nanocomposites because the network of filler improves the nanocomposites’ stress bearing ability and provides a high level of reinforcement. Many studies in the literature have demonstrated network formation in samples containing CNTs or clay^27–29^. Additionally, the immense surface area of large nanoparticles leads to the formation of a third interphase part in nanocomposites^30–34^. This phase is constructed because of robust communications at the interface between the polymer medium and the nanoparticles. Many models have considered the effects of the interfacial/interphase regions on the rigidity of various samples^35–40^. The interphase region can join the filler network, thereby facilitating the construction of large network in the system^41,42^. It can be said that the interphase region connects to the nanoparticles expanding the network. In the literature on the mechanical performance of HNT-based samples, HNT network and the interphase region have been neglected, even though they strongly influence the stiffness.

Kolarik model was used to estimate the tensile strength of HNT-filled composites in terms of HNT size, HNT fraction, and interphase levels^43^. Furthermore, Pukanszky’s equation was adapted to consider actual HNT concentration through the progressed Kolarik equation. Additionally, a few simple models were developed to estimate the strength of HNT-filled nanocomposites below or above the percolation onset^44,45^. However, the models for determining the modulus of such a system are limited. Ji proposed a model to determine the modulus of systems containing HNT networks and adjacent interphase zones^46^. In the present study, we focus on the Takayanagi model to approximate the modulus of HNT-filled nanocomposites by considering the interphase zones around dispersed and networked HNTs above the percolation onset.

Loos and Manas-Zloczower advanced the Takayanagi model to estimate the tensile modulus of CNT-filled samples by considering the dispersed and networked CNTs after the onset of percolation^47^. This advanced model can be applied to HNT systems because both CNTs and HNTs are tubular. In the present study, we advance this model to estimate the stiffness of HNT-based systems by considering the interphase zones around dispersed and networked HNTs. In addition, we present simple formulations for the percolation onset of HNTs, interphase content, effective HNT concentration, proportion of networked HNTs, and volume fractions of HNTs and interphase region in the net. The outputs of the innovative model are examined using the tentative data of many specimens from the relevant literature. Furthermore, the significances of all factors on the system modulus are determined to verify the proposed model. The proposed equations represent a simple methodology for approximating the stiffness of HNT-reinforced samples. The proposed model reveals the key factors governing the strengthening effects of nanoparticles and the interphase region in nanocomposites.

## Theoretical approaches

Loos and Manas-Zloczower extended the Takayanagi system by considering networked and dispersed CNTs in the system above the percolation onset^47^ as:

1 $$ E = \frac{{\varphi_{N} (1 - \varphi_{f} )E_{d} E_{N} + \varphi_{N} (\varphi_{f} - \varphi_{N} )E_{m} E_{N} + (1 - \varphi_{N} )^{2} E_{d} E_{m} }}{{(1 - \varphi_{f} )E_{d} + (\varphi_{f} - \varphi_{N} )E_{m} }}, $$

where $\varphi_{f}$ and $\varphi_{N}$ denote the volume fractions of filler and network, respectively. Moreover, E_d_, E_N_, and E_m_ denote the tensile moduli of the detached nanofiller, net, and polymer medium, respectively. The nonappearance of a net ($\varphi_{N}$ = 0) condenses this model to the following model:

2 $$ E = \frac{{E_{f} E_{m} }}{{(1 - \varphi_{f} )E_{f} + \varphi_{f} E_{m} }}. $$

Equation (1) can be applied to HNT-based systems because both HNTs and CNTs are tubular. However, this equation does not consider the interphase regions adjacent to the dispersed and networked HNTs. The interphase zone appears around both networked and dispersed nanoparticles. The interphase section is a separate phase near the nanoparticles, and it reinforces the sample. For this reason, the interphase section should be considered in Eq. (1), similar to the networked and dispersed nanofillers, because both the nanoparticles and the surrounding interphase zone reinforce the system simultaneously.

The concentrations and moduli of the interphase sections around the detached and networked nanoparticles are added to Eq. (1), similarly to the networked and dispersed nanoparticles, as:

3 $$ \frac{\begin{gathered} E = \frac{{\varphi_{N} (1 - \varphi_{f} )E_{d} E_{N} + \varphi_{N} (\varphi_{f} - \varphi_{N} )E_{m} E_{N} + (1 - \varphi_{N} )^{2} E_{d} E_{m} + \varphi_{iN} (1 - \varphi_{i} )E_{id} E_{iN} + \cdots }}{{(1 - \varphi_{f} )E_{d}^{{}} + (\varphi_{f} - \varphi_{N} )E_{m}^{{}} + \cdots }} \hfill \\ \varphi_{iN} (\varphi_{i} - \varphi_{iN} )E_{m} E_{iN} + (1 - \varphi_{iN} )^{2} E_{id} E_{m} \hfill \\ \end{gathered} }{{(1 - \varphi_{i} )E_{id} + (\varphi_{i} - \varphi_{iN} )E_{m} }}, $$

where $\varphi_{iN}$ denotes the volume fraction of the networked interphase zone, and $\varphi_{i}$ denotes the total volume fraction of the interphase region in the system. Moreover, E_id_ and E_iN_ denote the moduli of the interphase zones around dispersed and networked nanoparticles, respectively. This equation adequately reflects the reinforcing effects of HNTs and the interphase regions in the system.

Many researchers have mentioned that HNTs are tubular in real samples^17,48–52^; thus, it is assumed that HNTs have a cylindrical form in nanocomposites. Additionally, HNTs are non-uniform materials, and their properties greatly depend on the source material. Nevertheless, the proposed model is simple and comprehensive, and it considers the reinforcing efficiency of HNTs in the system by assuming the typical and simple features of HNTs. Moreover, various polymers are used in such systems, and the proposed model is suitable for all types of polymers.

Not all nanoparticles can contribute to the network after the onset of percolation. The percentage of a tubular nanofillers, such as HNTs building the network in a system is calculated^53^ as:

4 $$ f = \frac{{\varphi_{f}^{1/3} - \varphi_{p}^{1/3} }}{{1 - \varphi_{p}^{1/3} }}, $$

depending on the HNT concentration and percolation onset ($\varphi_{p}$).

The percolation onset of CNTs is associated with the CNT size and interphase depth^54^ as:

5 $$ \varphi_{p} = \frac{{\pi R^{2} l}}{{\frac{32}{3}\pi (R + t)^{3} \left[ {1 + \frac{3}{4}(\frac{l}{R + t}) + \frac{3}{32}\left( {\frac{l}{R + t}} \right)^{2} } \right]}}, $$

where “R” and “*l*” denote the radius and length of the nanoparticles, respectively, and “t” denotes the interphase depth. This equation can be used for HNT-based systems because both CNTs and HNTs have the same shape, and the onset of percolation depends on the filler shape and the nearby interphase section. It is clear that HNTs in a typical sample have a range of lengths, diameters, and shapes, but the average lengths and diameters of HNTs and their cylindrical shape are considered to simplify the calculations.

The entire volume portion of the interphase region in a system encompassing tube-shaped fillers such as HNT can be approximated^54^ as:

6 $$ \varphi_{i} = \varphi_{f} \left[ {\left( {1 + \frac{t}{R}} \right)^{2} - 1} \right], $$

which reflects the effects of HNTs and interphase dimensions, as well as HNT concentration, on the interphase concentration.

Both HNTs and the neighboring interphase sections reinforce the system. The effective HNT concentration is determined by considering the total amounts of reinforcements (HNTs and interphase regions) as:

7 $$ \varphi_{eff} = \varphi_{f} + \varphi_{i} = \varphi_{f} \left( {1 + \frac{t}{R}} \right)^{2} , $$

which correlates to the dimensions of HNTs and the interphase region, as well as HNT content.

The effective filler portion in the latter equation advances “f” (Eq. (4)) to:

8 $$ f = \frac{{\varphi_{eff}^{1/3} - \varphi_{p}^{1/3} }}{{1 - \varphi_{p}^{1/3} }}. $$

By using “f,” the volume fractions of HNTs and the interphase section in the network can be calculated as:

9 $$ \varphi_{N} = f\varphi_{f} , $$

10 $$ \varphi_{iN} = f\varphi_{i} . $$

When “f” from Eq. (8) is substituted into the latter equations, “$\varphi_{N}$” and “$\varphi_{iN}$” are extended as:

11 $$ \varphi_{N} = \frac{{\varphi_{eff}^{1/3} - \varphi_{p}^{1/3} }}{{1 - \varphi_{p}^{1/3} }}\varphi_{f} , $$

12 $$ \varphi_{iN} = \frac{{\varphi_{eff}^{1/3} - \varphi_{p}^{1/3} }}{{1 - \varphi_{p}^{1/3} }}\varphi_{i} , $$

which consider the HNT size, HNT concentration, interphase depth, and percolation onset when determining the concentrations of the networked HNT/interphase region.

All of the proposed equations define the main terms needed for modulus estimation by using the proposed model (Eq. (3)). These equations include meaningful and reasonable factors for HNTs and the nearby interphase regions.

The relative modulus is expressed as E/E_m_ by altering the proposed model (Eq. (3)) as:

13 $$ \frac{\begin{gathered} E_{R} = \frac{{\varphi_{N} (1 - \varphi_{f} )E_{d} E_{N} /E_{m} + \varphi_{N} (\varphi_{f} - \varphi_{N} )E_{N} + (1 - \varphi_{N} )^{2} E_{d} + \varphi_{iN} (1 - \varphi_{i} )E_{id} E_{iN} /E_{m} + \cdots }}{{(1 - \varphi_{f} )E_{d} + (\varphi_{f} - \varphi_{N} )E_{m} + \cdots }} \hfill \\ \varphi_{iN} (\varphi_{i} - \varphi_{iN} )E_{iN} + (1 - \varphi_{iN} )^{2} E_{id} \hfill \\ \end{gathered} }{{(1 - \varphi_{i} )E_{id} + (\varphi_{i} - \varphi_{iN} )E_{m} }}. $$

**Figure 1:**
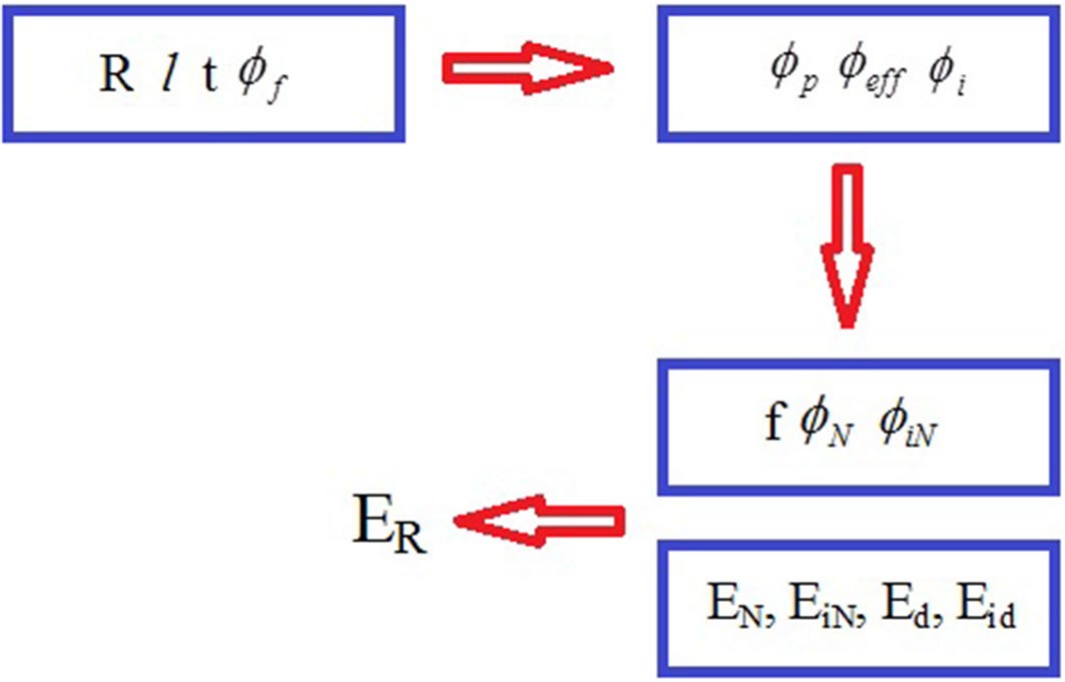
Schematic of the process of predicting the relative modulus by using Eq. (13) and various parameters and equations.

This expression reflects the influences of dispersed and networked HNTs and the adjacent interphase regions on the enhanced modulus of HNT-filled systems. Figure 1 schematically illustrates how to calculate the relative modulus by using Eq. (13) and the parameters and equations mentioned in this section.

## Results and discussion

### Model examination using tentative results

The predictions of the proposed model are assessed using the tentative moduli of real samples from relevant studies. The fine agreement between the predictions and the tentative results validate the correctness of the proposed model. Table 1 summarizes the characteristics of the samples analyzed herein, including starch/HNT^48^, polyamide 12 (PA12)/HNT^49^, poly(ε-caprolactone) (PCL)/HNT^50^, cellulose/HNT^51^, poly(lactic acid) (PLA)/HNT^17^, and linear low-density polyethylene (LLDPE)/HNT^52^. Experimental data were obtained from other relevant studies because our group possessed only one set of experimental data, which would have been inadequate to validate the proposed model. Information about the samples, including polymer strength and HNT dimensions, were obtained from the original references, as summarized in Table 1. However, the percolation onset was calculated using Eq. (5). Moreover, the moduli of the HNT network and the interphase region were calculated using the proposed model. The modulus of HNT is 140 GPa^55^. Furthermore, all data of the samples are available in the original papers, which are easily accessible. Additional data about the samples have not been included in Table 1 owing to space constraints. The morphological images of all samples highlight the cylindrical shape of the HNTs, which confirms this assumption. These data are then substituted in the proposed equations to calculate the relative modulus by using the advanced model.

**Table 1:** Real examples from valid articles, and estimation of various factors by using the developed equations.

No.	Samples [Ref.]	E_m_ (GPa)	R (nm)	*l* (μm)	t (nm)	$\varphi_{p}$	E_iN_ (GPa)	E_N_ (GPa)	E_id_ (GPa)	E_d_ (GPa)
1	Starch/HNT^48^	0.08	35	1.2	10	0.017	50	160	20	140
2	PA12^1^/HNT^49^	1.55	35	1.0	5.0	0.024	70	200	40	140
3	PCL^2^/HNT^50^	0.21	35	1.5	4.0	0.017	80	150	30	140
4	Cellulose/HNT^51^	1.80	25	2.0	8.0	0.008	80	270	30	140
5	PLA^3^/HNT^17^	2.90	40	1.2	12	0.019	60	225	25	140
6	LLDPE^4^/HNT^52^	0.20	30	1.3	2.0	0.018	30	147	15	140

^1^Polyamide 12.

^2^Poly(ε-caprolactone).

^3^Poly(lactic acid).

^4^Linear low density polyethylene.

**Figure 2:**
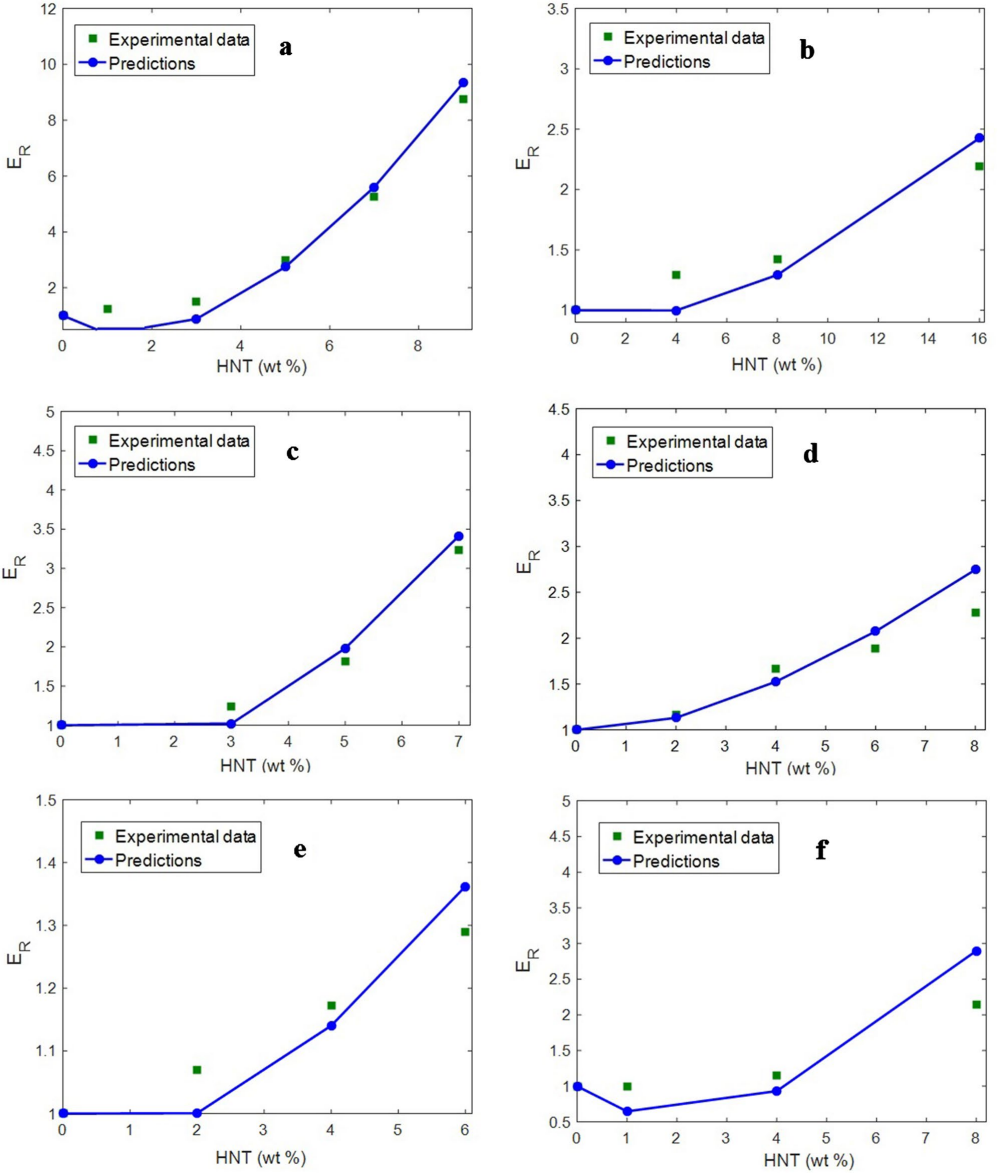
Experimental and theoretical (Eq. (13)) values of relative modulus of the (**a**) starch/HNT^48^, (**b**) PA12/HNT^49^, (**c**) PCL/HNT^50^ and (**d**) cellulose/HNT^51^, (**e**) PLA/HNT^17^, and (**f**) LLDPE/HNT^52^ systems.

Figure 2 shows the measured and calculated values of relative modulus for the samples considered herein. All calculations properly track the tentative data at all HNT concentrations. This fine agreement between the tentative and theoretical data validates the accuracy of the proposed model. Actually, the proposed model is verified using the experimental data of numerous samples. These samples include diverse polymers. For this reason, the proposed model can be applied to all types of polymers. The calculations of interphase and network parameters are summarized in Table 1. The interphase depth (t) ranged from 4 to 12 nm for the samples. This range is meaningful because it is as the nanoscale. The densest and the slimmest interphase sections are observed in cases of the PLA/HNT and PCL/HNT specimens, respectively. However, all results indicate that the samples contain interphase zones that contribute to the HNT network. HNT size and interphase depth are substituted in Eq. (5) to calculate the percolation onset. The cellulose/HNT sample exhibited the lowest percolation onset of 0.008, whereas PA12/HNT system exhibited the highest percolation onset of 0.024. These results reveal that network formation in the cellulose/HNT sample is faster than that in the PA12/HNT system. Clearly, HNT concentrations are higher than the percolation onsets, which indicate net formation in the samples.

Table 1 displays the modulus of the networked interphase zone in the samples. The E_iN_ values of the samples range from 30 to 80 GPa; the LLDPE/HNT sample has the poorest networked interphase section. Additionally, the HNT network moduli vary from 147 to 270 GPa, and the cellulose/HNT and LLDPE/HNT systems have the strongest and the weakest networks, respectively. These ranks indicate that the samples contain robust HNT networks. Moreover, the moduli of the interphase zones around the dispersed HNTs vary from 15 to 40 GPa for the samples. These estimates are logical because they are between the moduli of the polymer media (E_m_) and the HNTs (E_d_). All of these calculations are meaningful, which validate the proposed model for estimating the modulus of HNT-filled systems.

### Model validation based on parametric checks

Now, the effects of all factors on the moduli of the HNT samples are explained and justified to confirm the proposed model. We use contour plots to reveal the effects of two factors on the relative moduli considering normal values of the other factors. The middling levels in all calculations are as follows: R = 30 nm, *l* = 2 μm, $\varphi_{f}$ = 0.02, t = 10 nm, E_m_ = 2 GPa, E_d_ = 140 GPa, E_id_ = 30 GPa, E_N_ = 300 GPa, and E_iN_ = 80 GPa. These values represent the average levels, and the HNT dimensions in real samples may be smaller or larger owing to aggregation/agglomeration^56^. The contour plots are helpful for determining and optimizing the relative modulus by assuming important parameters. In fact, these plots reveal the main factors governing the stiffness of HNT-based systems.

**Figure 3:**
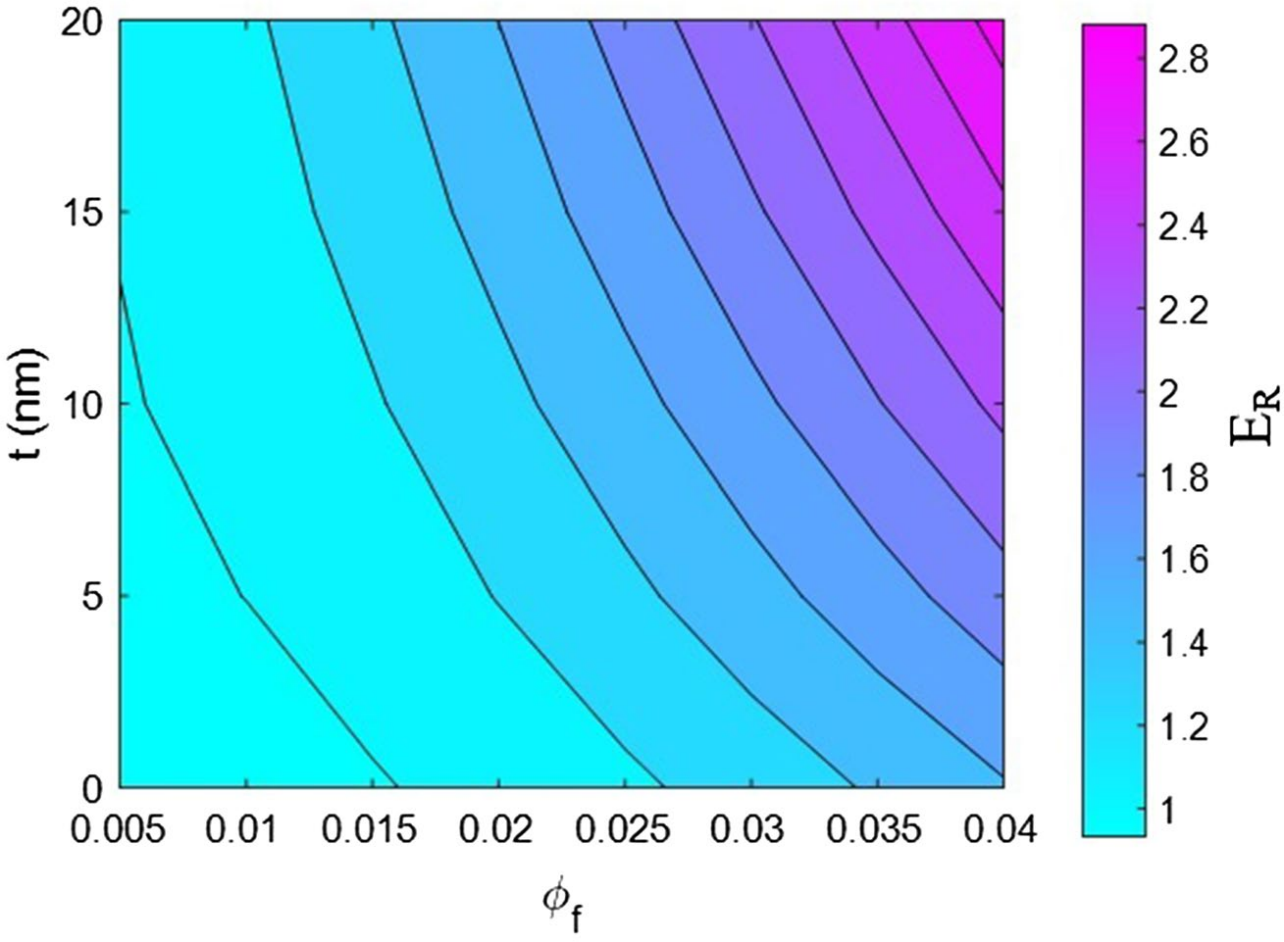
Dependences of “E_R_” on HNT concentration and interphase depth as estimated using the proposed model.

Figure 3 shows the relative modulus determined using the proposed model at various HNT concentrations and interphase depths. The relative modulus reaches 2.88 at the HNT volume fraction of 0.04 and interphase depth of 20 nm, but it decreases to approximately 1 at $\varphi_{f}$ < 0.01 and t < 5 nm. These values reveal that modulus of HNT system directly varies with both HNT concentration and interphase depth. High values of these factors significantly increase the modulus, but extremely low values of these factors ($\varphi_{f}$ < 0.01 and t < 5 nm) cannot stiffen the sample (relative modulus of 1). Consequently, a high HNT content and a dense interphase region are essential for increasing system stiffness.

A high concentration of HNTs undoubtedly enhances the system modulus because HNTs are significantly stiffer than the polymer medium. Owing to the presence of a large number of nanoparticles, the percolation onset is exceeded, and a network is created in the sample, which significantly increases system stiffness. Conversely, a low concentration of HNTs cannot provide adequate reinforcement because the HNTs cannot reinforce the immense expanse of the polymer medium. Moreover, an excessively low HNT concentration cannot stiffen the system because nanoparticles cannot create a network below the percolation onset. Accordingly, the system stiffness is directly correlated to HNT concentration, and the predictions of the established model are reasonable. The interphase depth has a significant effect on the relative modulus because a profuse interphase region has a strong reinforcing effect and leads to a desirable networking efficiency in the system. A profuse interphase region primarily reinforces the system because it is tougher than the polymer medium. Furthermore, a thicker interphase leads to the formation of a larger network in the system. By contrast, a narrow interphase has marginal reinforcing and networking effects, and it cannot produce a strong sample. The available models for the mechanics of various nanocomposites express the same trend between the extent of stiffening and the interphase depth^57,58^. These evidences confirm the predictability of the proposed model.

**Figure 4:**
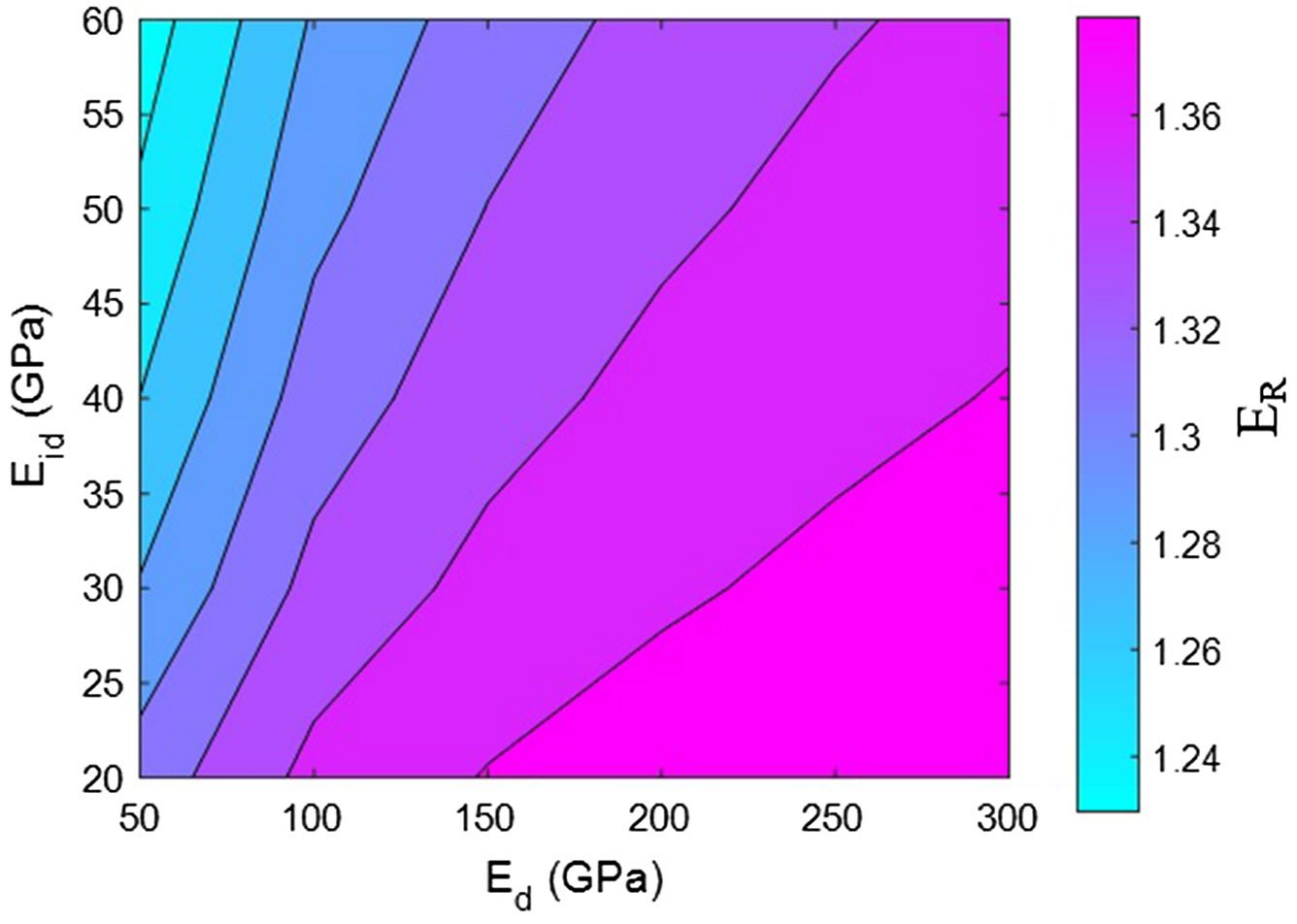
Calculations of relative modulus for various moduli of dispersed HNTs and adjacent interphase section.

Figure 4 portrays the disparity of relative modulus for various moduli of the dispersed HNTs and the adjacent interphase sections. The maximum relative modulus is achieved at the highest HNT modulus and the lowest interphase modulus. By contrast, the relative modulus decreases as the HNT modulus decreases and the interphase stiffness increases. Nevertheless, all ranges of the factors cause the relative modulus to change only from 1.23 to 1.38, which is rather marginal. Indeed, the effects of “E_d_” and “E_id_” on the modulus of HNT systems are insignificant and can be neglected.

The HNT modulus has a direct effect on the modulus of the entire system because the stiffer nanoparticles undoubtedly lead to the formation of a tough sample. Sturdier nanoparticles, such as HNTs, generally reinforce polymer nanocomposites because HNTs are considerably stiffer than the polymer medium. Consequently, it can be concluded that a stiffer component establishes a more solid system. Many models have considered the effect of nanofiller modulus on system stiffness^59,60^. Additionally, interphase modulus has a direct effect on the system modulus, but calculations reveal the adverse effect of interphase modulus on system stiffness. This is because of the considerably thinner interphase section (t = 10 nm) compared to the HNT radius (R = 30 nm). A thin interphase section reduces the volume fraction of the interphase in the samples, and consequently, its modulus cannot affect the sample modulus desirably. However, when the interphase depth is greater than the HNT radius, the modulus of the interphase section directly governs the modulus of the system because of the reinforcing effect of the interphase zone. This explanation demonstrates that the proposed system correctly predicts the effect of interphase modulus on the system stiffness.

**Figure 5:**
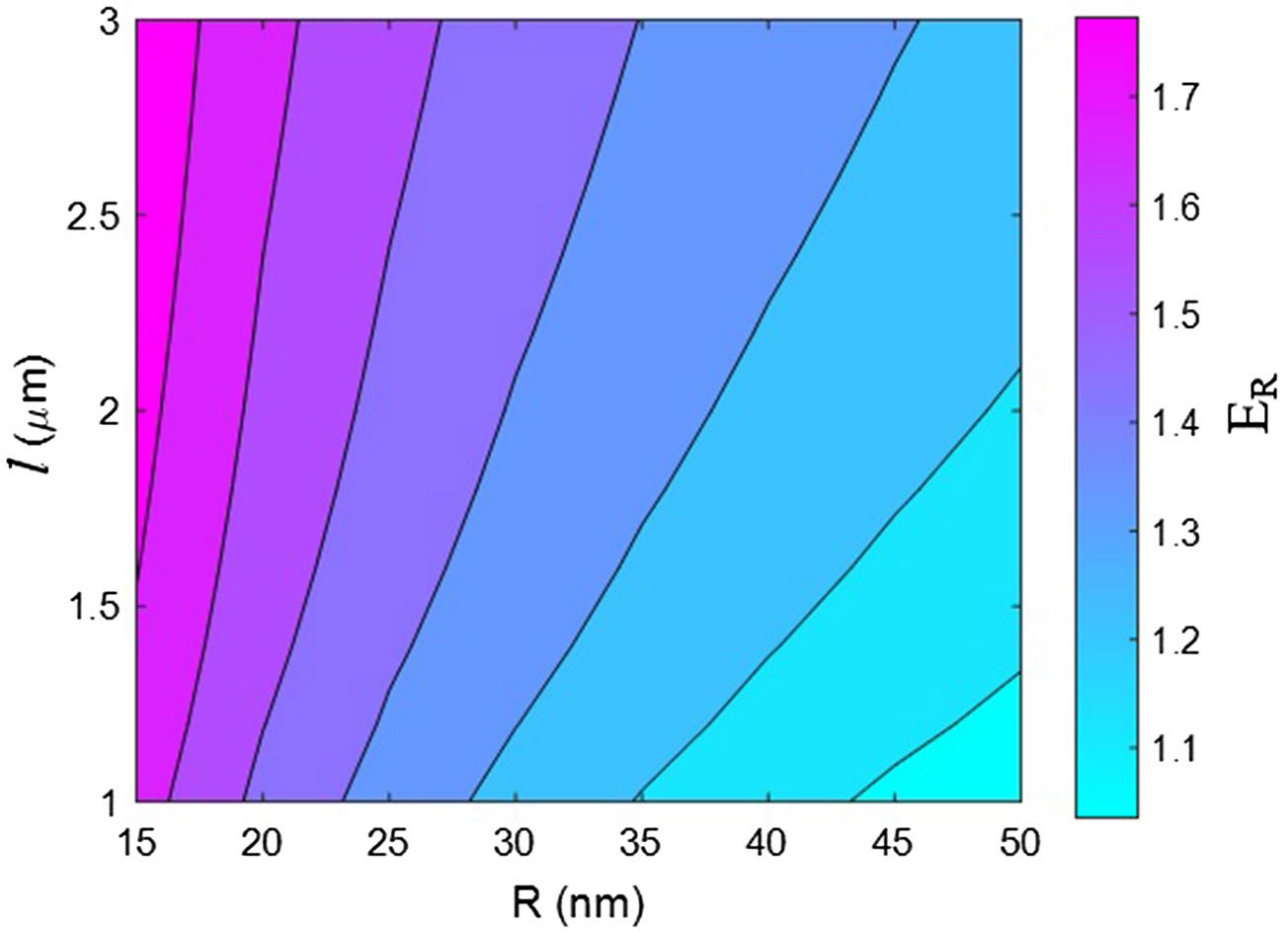
Effect of HNT size (radius and length) on the relative modulus according to the proposed model.

Figure 5 shows the relative moduli of the samples, as predicted using the proposed model, for various HNT sizes. The peak relative modulus of 1.77 is obtained at R = 15 nm and *l* > 1.7 μm, while the relative modulus decreases to 1.03 at R = 50 nm and *l* = 1 μm. Consequently, narrow and long HNTs are desirable for stiffening the system; thick and short HNTs have an insignificant reinforcing effect on the sample. HNT size is an important factor in the system because it changes after sample fabrication owing to accumulation^61,62^. This phenomenon warrants attention because HNT size is important for achieving a high level of reinforcement.

HNT radius governs the percolation onset, interphase concentration, and the concentrations of networked HNT and surrounding interphase section. Thin HNTs beneficially reduce the percolation onset, thereby widening the HNT networks and the interphase section. Narrow HNTs increase the concentration of the interphase section, thus strengthening the reinforcing effect of the interphase section. By contrast, thick HNTs increase the percolation onset and condense the network and interphase section. In fact, thick HNTs weaken the stiffening effect of the network and interphase region on the system, and thin HNTs considerably increase the system stiffness. These results validate the proposed model. In addition, longer HNTs reduce the percolation onset and increase the fractions of nanofillers and nearby interphase regions in the network because larger HNTs increase “f” (Eq. (8)). This means that longer HNTs lead to the formation of larger networks of HNTs and interphase regions. Conversely, shorter HNTs increase the percolation onset, which prevents the formation of large networks because a high percolation onset reduces the proportion of HNTs and interphase regions in the network (f). Consequently, large HNTs create a large network supporting a high stiffness, whereas short HNTs weaken the stiffening effect of the network. The existing models yielded the same relationship between filler size and nanocomposite stiffness^63–65^. These observations confirm the accuracy of the proposed model.

**Figure 6:**
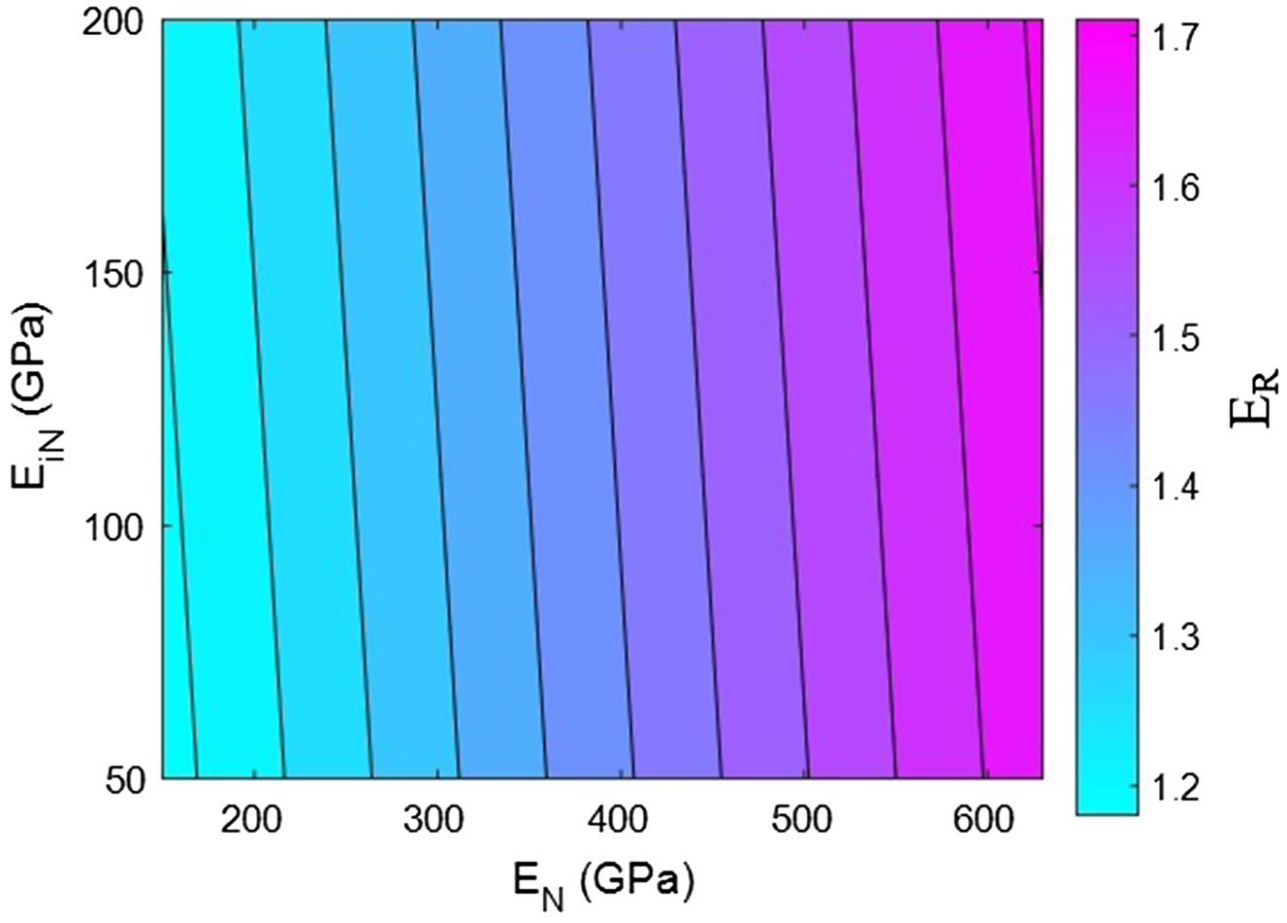
Variation of relative modulus at various moduli of the HNT network and the surrounding interphase zone according to the proposed model.

Figure 6 shows the relative moduli of the system at various moduli of the HNT network and the nearby interphase regions approximated using the proposed model. The low values of these parameters (E_N_ = 150 GPa and E_iN_ = 50 GPa) lead to a relative modulus of 1.18, but “E_R_” increases to 1.71 at E_N_ = 650 GPa and E_iN_ = 200 GPa. Therefore, the moduli of the HNT network and the nearby interphase regions directly govern the system stiffness. The higher values of these parameters yield a sturdier system, but their lower values reduce system stiffness. These results reveal the imperative effects of the HNT network and the adjoining interphase section on system stiffness.

The network modulus governs the stress loaded onto the sample because stress is shifted from the polymer medium to the nanoparticle network. Clearly, a stronger network tolerates more stress, whereas a weak network cannot bear a high load. Owing to the direct correlation between the stress bearing ability and stiffness of a sample, the modulus of the network directly governs the modulus of the entire system. This result is consistent with those reported in the literature^66,67^. Moreover, the modulus of the interphase section adjacent to the HNT network has a positive effect on system stiffness because the interphase region manages stress transfer. A stronger interphase can transfer a greater amount of stress from the polymer medium to the network, whereas a weak network breaks under loading. The logical effects of these parameters on system stiffness validate the proposed model.

**Figure 7:**
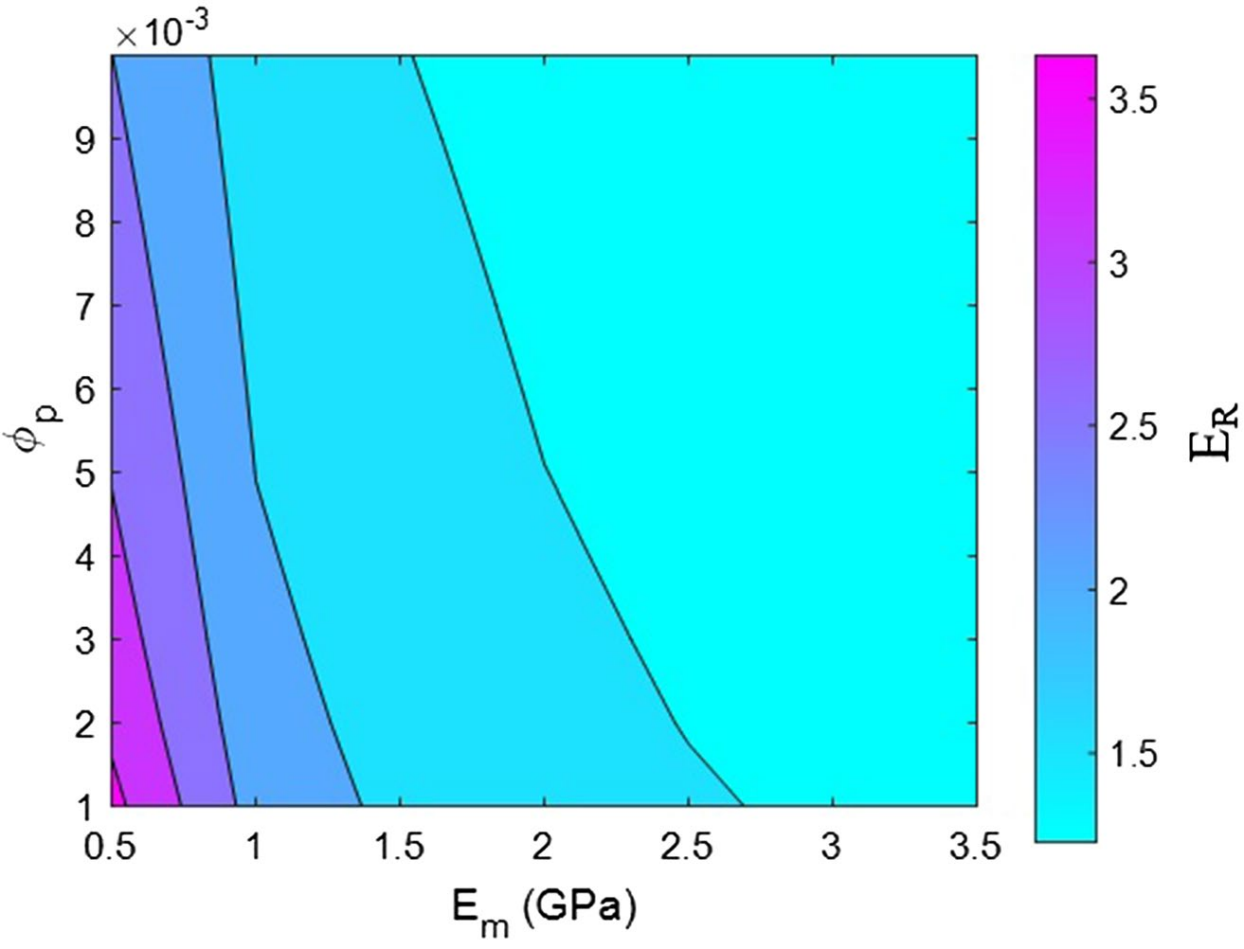
Estimates of relative modulus assuming various matrix moduli and percolation onset according to the current model.

Figure 7 displays the association of relative modulus with matrix modulus and percolation onset according to the proposed model. E_m_ = 0.5 GPa and percolation onset of 0.001 lead to a relative modulus of 3.7; nonetheless, “E_R_” meaningfully decreases to 1.2 when E_m_ > 2.7 GPa. Accordingly, low values of the matrix modulus and percolation onset are suitable for stiffening the system, but a high modulus of the polymer medium alone leads to a weak reinforcing effect. The wide ranges of relative modulus at various values of these factors signify that the matrix modulus and the percolation onset largely govern the system stiffness.

The modulus of the polymer medium inversely affects the modulus of the entire system owing to the extent of difference between the modulus of the polymer medium and that of nanoparticles. A large difference leads to a strong reinforcement effect, whereas a small difference weakens the reinforcing effect of the nanoparticles. Actually, when the moduli of the matrix and the nanoparticles are similar, the reinforcing effect of the nanoparticles is weak in composite systems. Consequently, the modulus of polymer media adversely affects the modulus of the entire system, and HNTs significantly reinforce weak polymer media. Moreover, percolation onset inversely affects the system stiffness, because a high percolation onset reduces the size of networked HNTs and the adjacent interphase section^67,68^. A high percolation onset decreases the fractions of networked HNTs/interphase section (f), but a low percolation onset increases the involvement of both HNTs and the interphase zone in the network. For this reason, a low percolation onset leads to the creation of a large network of HNTs and adjacent interphase zone, whereas a high percolation onset condenses the network. Based on these evidences, a low percolation onset increases system stiffness since the resulting larger network leads to a stiffer system. These findings reveal that the proposed model accurately predicts the effects of matrix modulus and percolation onset on system stiffness.

**Figure 8:**
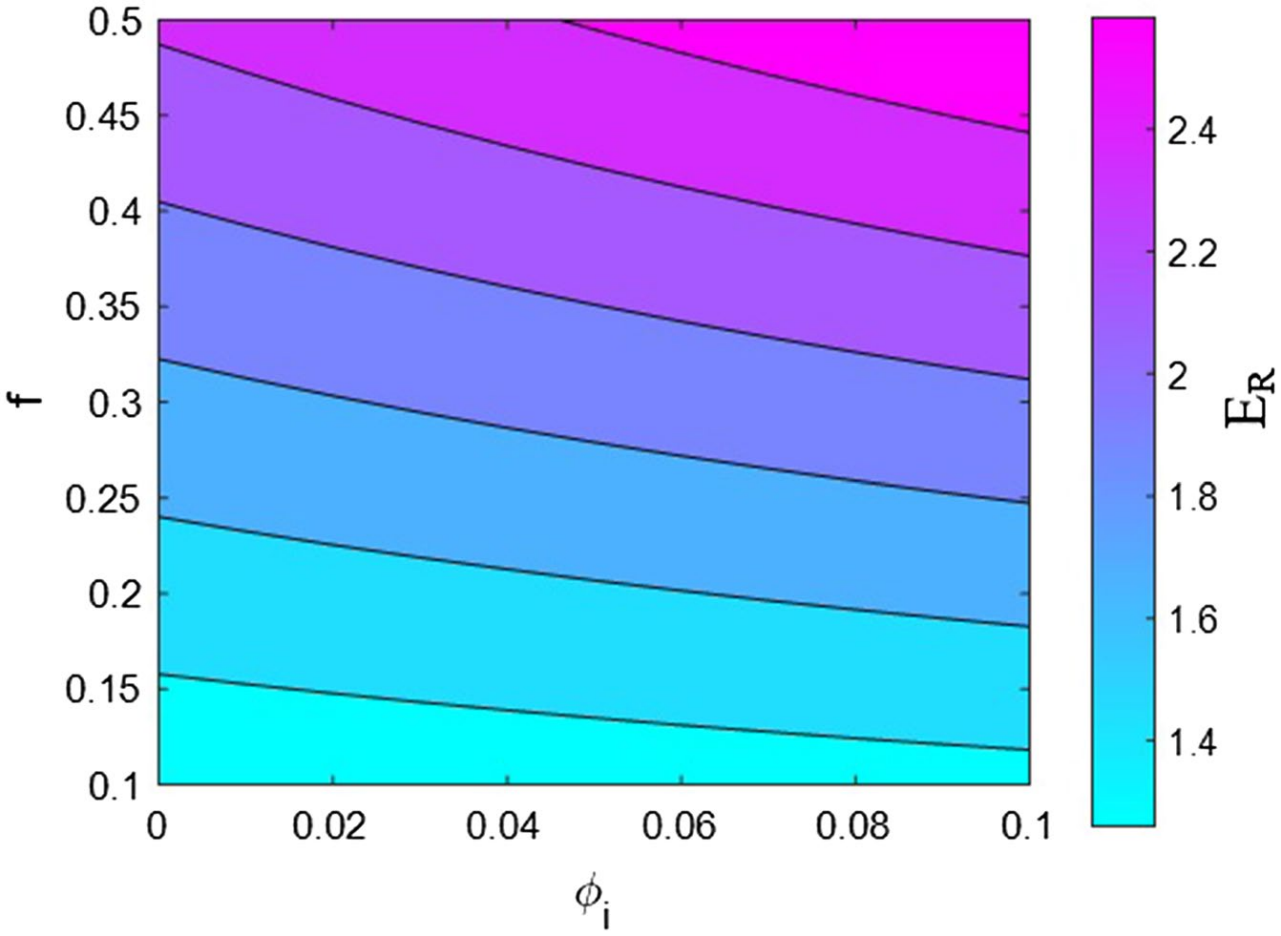
Effects of interphase content and proportion of networked HNTs/interphase section on the “E_R_” according to the proposed model.

Figure 8 exhibits the dependences of relative modulus on the interphase content and the proportion of networked HNT/interphase section according to the proposed model. An interphase concentration > 0.08 and f > 0.45 maximize the relative modulus to 2.6, while f < 0.12 decreases the relative modulus to 1.25. Accordingly, high levels of interphase concentration and network fraction positively affect the system modulus, but a low fraction of networked HNT/interphase section alone weakens the reinforcing effect. In other words, a small network has a marginal reinforcing effect on the system, but a large network with a high content of interphase zone produces a robust sample.

A large interphase zone provides a strong reinforcing effect because it increases the effective filler content and the network size. In this manner, a profuse interphase zone intensifies the reinforcing effect of HNTs. Moreover, a large interphase section swells the network stiffening the system owing to joining of the interphase sections with HNTs. In fact, a profuse interphase section intensifies the stiffening and networking efficiencies of HNTs in the samples, thus reinforcing the system. This evidence shows that the proposed model accurately predicts the association between relative modulus and interphase concentration. Furthermore, a higher level of stiffness can be expected with a higher fraction of networked HNTs/interphase section. Larger networks of HNTs and interphase sections have stronger reinforcing effects on the sample because they increase the stress bearing ability^66,69^. By contrast, a low fraction of networked HNTs/interphase section leads to a small network, which can bear a lower amount of stress and fails under stress loading. Accordingly, a system with a small network has low stiffness, and the direct dependence of the reinforcing effect on the network fraction is rational, which validates the proposed model.

## Conclusions

The developed Takayanagi model for estimating the stiffness of CNT-filled system was modified to approximate the modulus of polymer HNT samples by considering the interphase zones adjacent to dispersed and networked HNTs. The outputs of the proposed equations were consistent with the experimental data of numerous real examples. Moreover, the calculations for the interphase and network features were meaningful, thus validating the proposed model. The relative modulus was 2.88 at the HNT volume fraction of 0.04 and interphase depth of 20 nm, but $\varphi_{f}$ < 0.01 and t < 5 nm could not reinforce the system. The best reinforcing effect was achieved at the highest HNT modulus and the lowest interphase modulus, but these factors negligibly altered the relative modulus from 1.23 to 1.38. Narrow and long HNTs were able to reinforce the system, but thick and short HNT were unable to do so. Moreover, the moduli of the HNT network and the adjacent interphase section directly governed the sample stiffness. Low values of the matrix modulus and percolation onset were appropriate from the viewpoint of reinforcement, but only a high modulus of the polymer medium led to poor stiffening. These factors had the most significant effects of the system modulus. The maximum relative modulus of 2.6 was achieved when the interphase concentration > 0.08 and network proportion > 0.45, but when the network fraction was 0.12, E_R_ decreased to 1.25. Consequently, large values of interphase concentration and network fraction improved the system modulus.
